# Transcriptome Analysis Reveals Candidate Genes Related to Color Fading of ‘Red Bartlett’ (*Pyrus communis* L.)

**DOI:** 10.3389/fpls.2017.00455

**Published:** 2017-03-31

**Authors:** Zhigang Wang, Hong Du, Rui Zhai, Linyan Song, Fengwang Ma, Lingfei Xu

**Affiliations:** College of Horticulture, Northwest A&F UniversityYangling, China

**Keywords:** ‘Red Bartlett’ pear, transcriptome, color fading, anthocyanin, differentially expressed genes

## Abstract

The red color of fruit is an attractive plant trait for consumers. Plants with color-faded fruit have a lower commercial value, such as ‘Red Bartlett’ pears (*Pyrus communis* L.) that have dark-red fruit in the early stages of fruit development that subsequently fade to red-green at maturity. To identify the reason for color fading, we first analyzed the anthocyanin content of peel from ‘Red Bartlett,’ which displays the color fading phenomenon, and ‘Starkrimson,’ which has no color fading. Results showed that the anthocyanin content of ‘Red Bartlett’ peel decreased significantly late in fruit development, while in ‘Starkrimson’ there was no significant decrease. Next, RNA-Sequencing was used to identify 947 differentially expressed genes (DEGs) between ‘Red Bartlett’ and ‘Starkrimson.’ Among them, 471 genes were upregulated and 476 genes were downregulated in ‘Red Bartlett’ at the late development stage relative to ‘Starkrimson.’ During ‘Red Bartlett’ color fading, the structural gene *LDOX* and six GST family genes were downregulated, while FLS, LAC, POD, and five light-responding genes were significantly upregulated. Additionally, 45 genes encoding transcription factors MYB, bHLH, WRKY, NAC, ERF, and zinc finger were identified among 947 DEGs. Changes in the expression of these genes may be responsible for the decrease in anthocyanin accumulation in ‘Red Bartlett’ fruit. Taken together, this study demonstrated that color fading of ‘Red Bartlett’ was closely linked to reduced anthocyanin biosynthesis, increased anthocyanin degradation and suppression of anthocyanin transport. It also provided novel evidence for the involvement of light signals in the color fading of red-skinned pears.

## Introduction

Fruit peel color is a key trait for fruit quality. In red fruit, the key factor affecting fruit peel coloration is the plant pigment anthocyanin. Anthocyanin is a water-soluble flavonoid and a natural colorant that accumulates widely in many plant tissues such as the flesh (Boss et al., 1996), peel (Honda et al., 2002), and petal (Vaknin et al., 2005).

The anthocyanin biosynthetic pathway has been described in a number of model plants (Winkel-shirley, 2001) and also in many fruit trees such as apple (Espley et al., 2007), grape (Kobayashi et al., 2002), and pear (*Pyrus communis* L.) (Fischer et al., 2007). In pear, the structural genes involved in anthocyanin biosynthesis have been isolated and are coordinately regulated by a MBW complex of MYB, basic helix-loop-helix proteins (bHLH), and WD40 proteins. For example, MYB10 and PyMYB10.1 Interact with bHLH, Enhance Anthocyanin Accumulation in Pears (Feng et al., 2015). By regulating transcript levels of PcUFGT, the methylation level of the PcMYB10 promoter affected the formation of the green-skinned sport of ‘Red Bartlett’ (Wang et al., 2013). PbMYB9, a *TRANSPARENT TESTA 2*-type MYB transcription factor (TF) regulating the flavonol branch, has also been identified in pear fruit (Zhai et al., 2015). In apple, the cold-induced bHLH TF gene MdbHLH3 promoted fruit coloration (Xie et al., 2012). Recently, it has been reported that anthocyanin biosynthesis was also regulated by other TFs such as WRKY and NAC family members (Johnson et al., 2002; Zhou et al., 2015). In addition to the effects of endogenous genes, anthocyanin synthesis was also modulated and affected by environmental factors, in particular by light (Cutuli et al., 1999). When dark-grown fruit were exposed to sunlight, MdMYB1 transcript levels increased over several days, correlating with anthocyanin synthesis in red apple (Takos et al., 2006). Furthermore, the associated MBW complex could be decreased under low light intensity and dark conditions, leading to the downregulation of structural genes and a resulting decrease in anthocyanin content (Azuma et al., 2012). In addition, AP2 and WARK regulated the anthocyanin biosynthesis in red skinned ‘Starkrimson,’ and ANR and LAR promote PA biosynthesis and contribute to the green skinned variant (Yang et al., 2015a). Recently, reports of anthocyanin degradation and color fading have received much attention. Three common enzyme families, polyphenol oxidases (PPOs), class III peroxidases, and β-glucosidases, were reported to be involved in anthocyanin degradation (Oren-Shamir, 2009). To date, BcPrx01, a basic vacuolar peroxidase, was shown to be responsible for anthocyanin degradation in Brunfelsia calycina flowers (Zipor et al., 2015) while laccase, a novel anthocyanin degradation enzyme, was identified in litchi pericarp (Fang et al., 2015).

However, anthocyanin content was not only associated with anthocyanin metabolism but also with anthocyanin transport. Anthocyanins were synthesized at the cytoplasmic face of the endoplasmic reticulum before being transported to the vacuole for anthocyanin accumulation (Marrs et al., 1995). Moreover, it was described that in addition to the glutathione *S*-transferase (GST) family, the ATP-binding cassette (ABC) and multidrug and toxic compound extrusion (MATE) families were also involved in anthocyanin transport (Mueller et al., 2000; Klein et al., 2006; Gomez et al., 2009). In maize, the *Bronze2* (*bz2*) gene, a GST family member, was confirmed to be involved in vacuolar transfer of anthocyanins by conjugating with glutathione (Marrs et al., 1995). *TRANSPARENT TESTA 19* (*TT19*), a member of the *Arabidopsis thaliana* GST gene family, was also found to be required for the transportation of anthocyanins into vacuoles (Kitamura et al., 2004). MdGST (MDP0000252292) from apple has been shown to be the most suppressed gene in a yellow-skin somatic mutant apple line with corresponding reductions in anthocyanin content (Elsharkawy et al., 2015). Nevertheless, to date, no direct evidence for the role of GSTs in conjugating anthocyanins in pear has been found.

In this study, utilizing RNA-sequencing (RNA-Seq) technology, we sequenced the transcriptome of two red-skinned European pears: ‘Red Bartlett,’ the fruit of which were red in the early stages of development before obviously fading into red-green during the later development period, and ‘Starkrimson,’ the fruit of which lacked clear color deviation and maintained a purplish-red color throughout whole development. Also, we identified a set of differentially expressed genes (DEGs) potentially involved in fruit coloration.

## Materials and Methods

### Plant Materials and Fruit Treatment

**FIGURE 1:**
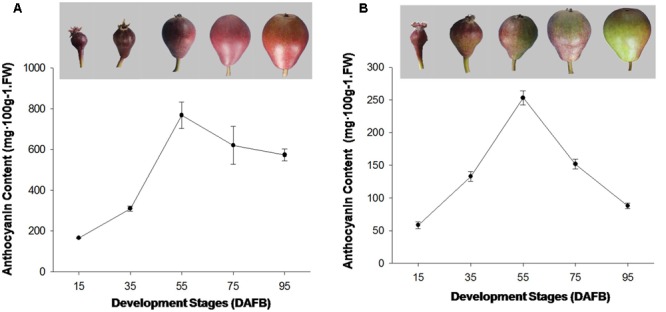
**(A)** Coloration and anthocyanin content of ‘Starkrimson’ pear peel during fruit development. **(B)** Coloration and anthocyanin content of ‘Red Bartlett’ fruit peel during development.

In this study, the fruit of ‘Red Bartlett’ and ‘Starkrimson’ were selected as plant materials and were obtained from the orchard of Mei County, Shanxi Province, China, in 2015. The fruits of pear were harvested at 15, 35, 55, 75, and 95 days after full bloom (DAFB) (as showed in **Figure 1**). The peel of the each pears was pared at a thickness of approximately 1 mm, frozen immediately in liquid nitrogen, and then stored at -80°C for further study.

### Extraction and Determination of Anthocyanins

Anthocyanin extraction was conducted according to the method of Giusti and Wrolstad (2001), with slight modifications. Approximately, 1 g of skin tissue was collected and quickly ground into powder in liquid nitrogen before 5 ml of 1% HCL-methanol solution was added and the sample was incubated in the dark at 4°C for 12 h. After centrifugation at 12,000 × *g* for 20 min, the supernatant was transferred to a clean tube and used for two dilutions, one with 0.025 M potassium chloride buffer (pH = 1.0) and the other with 0.4 M sodium acetate buffer (pH = 4.5). These dilutions were left to equilibrate for 15 min before the absorbance of each dilution was measured at 530 and 700 nm with a UV-Visible spectrophotometer (UV-1700, Kyoto, Japan), using a blank cell filled with distilled water for calibration. The anthocyanin content was calculated using the following formula:

$$\begin{array}{c} C\;=\;A\;\times \;V\;\times \;n\;\times \;\mathrm{MW}\;\times \;100/(\varepsilon \;\times \;m), \end{array}$$

where C stood for anthocyanin content (mg⋅100 g^-1^ FW), V for extraction solution volume, n for dilution factor, MW for the molecular weight of cyanidin-3-glucoside: 449.2, 𝜀 for molar absorptivity: 30200, m for the weight of fruit skin, and A = (A530–A700 nm) pH_1.0_ (A530–A700 nm) pH_4.5_. The value for each sample represented the mean of three independent biological replicates.

### RNA Extraction and cDNA Synthesis

Owing to anthocyanin levels are peaked at 55 DAFB in both of ‘Red Bartlett’ and ‘Starkrimson,’ five fruit peels of ‘Red Bartlett’ and ‘Starkrimson’ at 35 and 75 DAFB were mixed respectively and used for RNA-sequencing, with two biological replicates used for each cultivar at each time point. The total RNA was extracted and purified using an RNAprep Pure Plant Kit (TIANGEN, Beijing, China), according to the manufacturer’s instructions. RNA quality was checked using a NanoDrop Spectrophotometer (NanoDrop 2000C, Wilmington, DE, USA) and 5 μl of RNA was used in 1–1.5% agarose gel electrophoresis to examine its integrity and purity. First strand cDNA synthesis was performed using a PrimeScript RT-PCR Kit (TaKaRa, Dalian, China) according to the manufacturer’s instructions and stored at -20°C for RT-qPCR assays.

### Library Construction and Transcriptome Sequencing

The Agilent 2100 Bioanalyzer was used to further examine the RNA quality of all samples. Samples with an RNA integrity number ≥7, RNA content of ≥4 μg, an RNA concentration of ≥50 ng/μl, and 28S:18S RNA ratio of ≥2 were used to construct RNA-Seq libraries for Illumina sequencing. Library generation involved five steps: the first step was to purify and fragment the mRNA; next, double stranded cDNA was synthesized using the fragmented mRNA, third, the sticky end of short fragments was repaired with end repair reagents to avoid self-connection; fourthly, sequencing adaptors were added to the cDNA fragments that were then enriched by PCR amplification; and finally, quality control analysis of the constructed libraries was carried out. The libraries were constructed using an Illumina HiSeq^TM^ 2500 by the Millennium Corporation (Shenzhen, China). Including the two developmental stages of the two European pears, four RNA-Seq libraries were constructed and labeled as follows: Starkrimson-35 (35 DAFB of ‘Starkrimson’), Red Bartlett-35 (35 DAFB of ‘Red Bartlett’), Starkrimson-75 (75 DAFB of ‘Starkrimson’), and Red Bartlett-75 (75 DAFB of ‘Red Bartlett’).

### RNA-Sequencing Data Analysis

To ensure the accuracy and reliability of RNA-sequencing data, some poor quality reads were eliminated from the raw reads and only the remaining high-quality reads (clean reads) were used for statistics analysis. The level of gene expression was determined according to the number of fragments per kilobase of exon per million fragments mapped. The genes with a false discovery rate of <0.001 and an absolute value of the log_2_ (Fold Change) ≥1 were defined as DEGs. The functional annotation information for these DEGs was obtained using Batch Entrez^1^. Additionally, Gene Ontology (GO) annotations and Kyoto Encyclopedia of Genes and Genomes (KEGG) pathway analyses were conducted using Blast2GO software (Conesa et al., 2005), and provided a comprehensive set of evidenced-based associations between the genes and UniProtKB proteins or the significantly enriched pathways (Mao et al., 2005; Dimmer et al., 2011).

### RT-qPCR Validation

In order to verify the reliability of the RNA-Seq results, eight important DEGs were selected and measured by RT-qPCR on an iQ5 (Bio-Rad, Berkeley, CA, USA) using the SYBR Premix Ex Taq II (TaKaRa) according to the manufacturer’s instructions. *ACTIN* was used as the reference gene, and the relative gene expression levels were determined using the 2^-ΔΔCT^ approach. Each sample (including three biological repetitions) was quantified in triplicate. The reaction system included 100 ng cDNA, 0.8 μl forward and reverse primers (10 μM), respectively, and 10 μl SYBR Premix Ex Taq II, adjusted to 20 μl with sterile water. A two-step program was used, with an initial hot start at 95°C for 30 s followed by 40 cycles of 95°C for 5 s and 60°C for 34 s. Melting curves were generated using the following program: 95°C for 15 s, 60°C for 1 min, and 95°C for 15 s. A list of RT-qPCR primers for *ACTIN* and the 12 selected genes is displayed in **Supplementary Table S1**.

### Statistical Analysis

Statistical analysis was performed using Microsoft Excel 2010 and Sigmaplot 10.0 (Systat Software, Inc., San Jose, CA, USA). Each value represented mean ± SD of three independent biological replicates.

## Results

### Changes in the Anthocyanin Content and ACS1 Gene Expression of the Two Cultivars

The red color appeared on fruit at the early development stages in ‘Red Bartlett.’ The coloration reduced from the middle stages (DAFB 55) as the fruit matured before finally most of the fruit surface turned green. The anthocyanin content of ‘Red Bartlett’ fruit increased significantly at the early stage, reached 253 mg⋅100 g^-1^ FW at 55 DAFB before subsequently declining to 88 mg⋅100 g^-1^ FW at 95 DAFB, in accordance with the color fading of ‘Red Bartlett’ (**Figure 1A**). However, in ‘Starkrimson’ fruit, the coloration progressively changed from red to dark-red until 55 DAFB and then appeared bright red after 55 DAFB. Furthermore, the anthocyanin content of ‘Red Bartlett’ increased sharply and significantly during the early stages of development, before decreasing after 55 DAFB. This differed from ‘Starkrimson’ where the anthocyanin levels did not change significantly after 55 DAFB (**Figure 1B**). Moreover, the anthocyanin level of ‘Starkrimson’ was always higher than ‘Red Bartlett’ in whole development stage.

The expression of ACS1 gene in both cultivars were similar and showed the same change trend (Supplementary Figure S1), indicating that the developmental stages under comparison were homogeneous between the two cultivars.

### The RNA-Seq Data Analysis

To ensure the validity of transcriptome results, the data obtained from RNA-Seq was used for statistical analysis. In the present study, the high-quality libraries (with a mapping rate higher than 56% and Q20 and Q30 values higher than 90%), were constructed using the fruit skin of Starkrimson-35 (35 DAFB of ‘Starkrimson’), Red Bartlett-35 (35 DAFB of ‘Red Bartlett’), Starkrimson-75 (75 DAFB of ‘Starkrimson’), and Red Bartlett-75 (75 DAFB of ‘Red Bartlett’), respectively (**Supplementary Table S7**). Additionally, the gene coverage coincided with the results of the library construction (Supplementary Figure S2). These results indicate that the RNA-Seq data we obtained was useable for this study.

### Identification of DEGs between ‘Red Bartlett’ and ‘Starkrimson’

**FIGURE 2:**
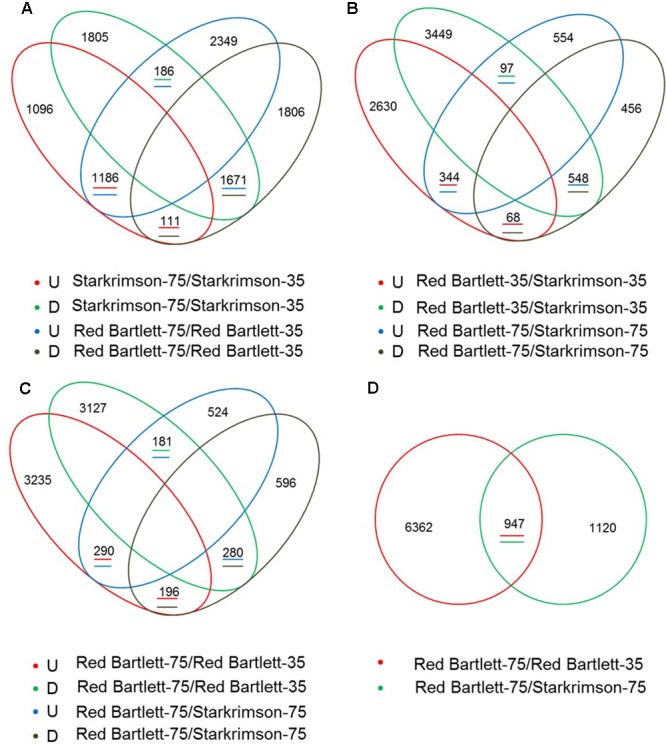
**Venn diagram of the differentially expressed genes (DEGs) screened by RNA-Seq analysis during two fruit developmental stages (35 DAFB and 75 DAFB) in two cultivars (‘Red Bartlett’ and ‘Starkrimson’).** The letters U and D represent upregulation and downregulation, respectively. **(A)** Quantity of upregulated or downregulated DEGs between two stages in two cultivars. **(B)** Quantity of upregulated or downregulated DEGs between two pears at two stages. **(C)** Quantity of upregulated or downregulated DEGs between two cultivars at 75 DAFB, and quantity of upregulated or downregulated DEGs between two stages in ‘Red Bartlett.’ **(D)** number of DEGs between the two stages in two cultivars.

To find candidate genes, pairwise comparison analysis was conducted. In the two cultivars, 3154 redundant DEGs (with an absolute value of log_2_ (Fold Change) ≥1 and a false discovery rate of <0.001) were identified between 75 DAFB and 35 DAFB (**Figure 2A**). At the two developmental stages, 1057 redundant DEGs were also found between the two cultivars (**Figure 2B**). Overall, by pairwise comparison, 947 DEGs were redundant between ‘Red Bartlett-75/Red Bartlett-35’ (DEGs between the two development stages in ‘Red Bartlett’) and ‘Red Bartlett-75/Starkrimson-75’ (DEGs between the two cultivars at 75 DAFB) (**Figure 2D**). Compared with ‘Starkrimson,’ 471 DEGs were significantly upregulated at 75 DAFB in ‘Red Bartlett,’ while 476 were significantly downregulated (**Figure 2C**). These 947 DEGs were, therefore, selected as candidate genes potentially associated with the color fading of ‘Red Bartlett’ (**Supplementary Table S2**), and were subjected to further functional analysis.

### GO Annotation, KEGG Pathway, and Enrichment Analyses

**FIGURE 3:**
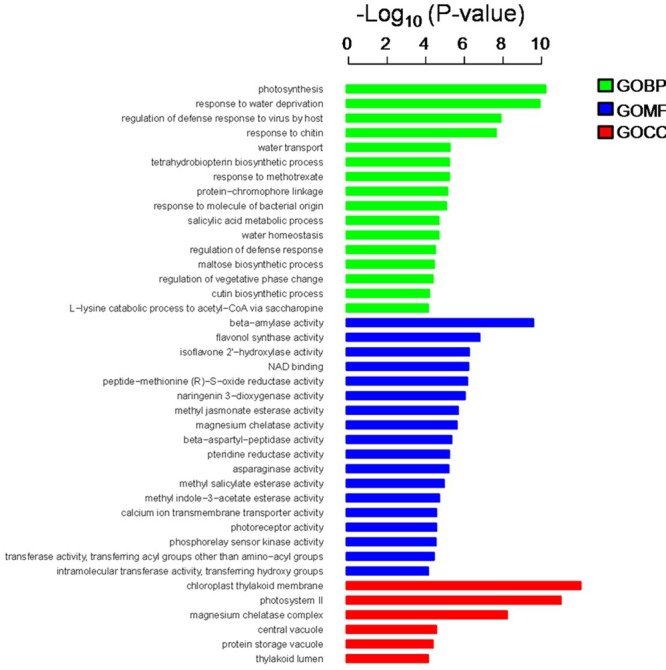
**Gene ontology (GO) annotation and enrichment analysis of 947 DEGs.** The horizontal axis shows the negative log_10_ of the *p*-value, while the vertical axis represents biological process, molecular function, and cellular component, respectively.

In the present study, the predicted functions of 947 DEGs were obtained by GO annotation, KEGG pathway, and enrichment analyses. According to GO annotation, these DEGs were distributed into 40 functional terms as follows: 16 terms for biological process; 18 terms for molecular function, and six terms for cellular component. The genes in the biological process group were mainly involved in photosynthesis and regulation of defense response. The molecular function terms related to beta-amylase, flavonol synthase (FLS), and isoflavone 2′-hydroxylase. Most of the cellular component genes were located in the chloroplast thylakoid membrane, photosystem II, magnesium chelatase complex, and vacuole (**Figure 3**). Meanwhile, KEGG pathway and enrichment analysis showed that DEGs were significantly enriched in the pathways of circadian rhythm, photosynthesis, and peroxisome (**Supplementary Table S3**).

### Analysis of Genes Involved in the Anthocyanin Synthesis, Degradation, and Transport

**FIGURE 4:**
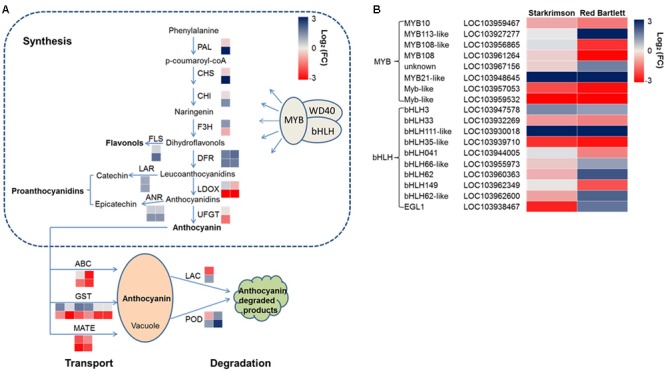
**(A)** Expression pattern of genes involved in anthocyanin synthesis, degradation, and transport. **(B)** Expression pattern of MYB and bHLH TFs involved in the regulation of anthocyanin synthesis. The value of log_2_ [fold change (FC)] was represented using the depth of color, with blue representing upregulation and red representing downregulation. Fold change means the ratio of fragments per kilobase of exon per million fragments mapped between the two stages in two cultivars. Small squares in the upper line represent the Log_2_ value of ‘Starkrimson,’ while the lower line represents the Log_2_ value of ‘Red Bartlett,’ with a square in the same line representing one copy of each gene.

Structural genes involved in anthocyanin synthesis were identified (**Figure 4A** and **Supplementary Table S4**). The predicted proteins encoded by upstream genes included one phenylalanine ammonia lyase (LOC103962533), one chalcone synthase (LOC103959489), one chalcone isomerase (LOC103940646), and one flavanone-3-hydroxylase (LOC103953484). The predicted proteins encoded by downstream genes included two dihydroflavonol-4-reductase (LOC103928717 and LOC103954960), two leucoanthocyanidin dioxygenase (LDOX) (LOC103958614 and LOC103966324) and one UDP-glucose:flavonoid 3-*O*-glucosyltransferase (LOC103951514). In ‘Red Bartlett,’ the predicted coding genes of the phenylalanine ammonia lyase (LOC103962533), chalcone synthase (LOC103959489), and dihydroflavonol-4-reductase (LOC103928717 and LOC103954960) were significantly upregulated at 75 DAFB, while two LDOX-predicted coding genes (LOC103958614 and LOC103966324) were significantly downregulated. Moreover, the UFGT-predicted coding gene (LOC103951514), a key anthocyanin synthetic gene, decreased in expression during both development stages in ‘Red Bartlett’ but did not change in expression in ‘Starkrimson.’ Genes related to other flavonoid metabolic pathways showed a significant increase in mature ‘Red Bartlett’ fruit, especially the FLS-predicted coding gene (LOC103933697).

In the present study, one LAC-predicted coding gene (LOC103946137) and two POD-predicted coding genes (LOC103945527 and LOC103961180) were identified (**Figure 4A** and **Supplementary Table S4**). In ‘Red Bartlett,’ expression levels of these three genes at 75 DAFB were all higher than at 35 DAFB. This result suggests that these genes involved in anthocyanin degradation may take part in the color fading phenotype of ‘Red Bartlett.’

Ten predicted coding genes involved in anthocyanin transport were identified (**Figure 4A** and **Supplementary Table S4**), including six GST (LOC103955362, LOC103945951, LOC103945952, LOC103955337, LOC103960192, and LOC103960208), two MATE (LOC103933301 and LOC103955660), and two ABC (LOC103928557 and LOC103947696). Moreover, two genes predicted to encode ABCs were significantly downregulated in expression in the late fruit development stage of ‘Red Bartlett.’ However, expression of these two genes did not significantly differ [the absolute value of log_2_ (Fold Change) <1] between the two cultivars at 75 DAFB. Therefore, six GSTs and two MATEs, but not the two ABCs, were candidate key genes for anthocyanin transport.

Anthocyanin synthesis was regulated by a series of TFs and in the present study, 45 DEGs predicted to encode TFs were identified (**Figure 4B** and **Supplementary Table S5**). These genes were divided into six categories: MYB, bHLH, WRKY, NAC, ethylene response factor (ERF), and zinc finger. Among these 45 genes, 20 have significantly increased transcript levels at 75 DAFB in ‘Red Bartlett’ relative to 35 DAFB, while expression of the remainder decreased significantly. Genes annotated as MYB 108 (LOC103961264), MYB 108-like (LOC103956865), Myb-like (LOC103959532 and LOC103957053), bHLH149 (LOC103962349), bHLH041 (LOC103944005), and bHLH35-like (LOC103939710) were downregulated in the mature fruit of ‘Red Bartlett’ while the rest of genes predicted to encode MYB and bHLH TFs were upregulated (**Figure 4B**). Additionally, TFs annotated as WRKY, NAC, ERF, and zinc finger family members also changed significantly in expression during fruit development in ‘Red Bartlett’ (**Supplementary Table S5**). Finally, seven DEGs involved in the light signal transduction pathway and photomorphogenesis were also identified. These genes were annotated as UV RESISTANCE LOCUS8 (LOC103954102 and LOC103962736), phytochrome B (LOC103961302 and LOC103928909), Phytochrome-interacting factors 3 (LOC103955304), CONSTITUTIVELY PHOTOMORPHOGENIC 1-like (COP1) (LOC103960466) and SUPPRESSOR OF PHYA105 (SPA; LOC103965891). In ‘Red Bartlett,’ the transcript level of these seven genes increased significantly at the late development stage (**Supplementary Table S6**).

Therefore, these results demonstrate that the color fading phenotype of ‘Red Bartlett’ was induced by anthocyanin synthesis, degradation, and transport, as well as by TF regulation. They also indicate that the light signal was involved in color fading of ‘Red Bartlett’ pears. To verify the reliability of the RNA-Seq data, 12 candidate DEGs were selected for RT-qPCR assays. The results of RT-qPCR were consistent with those of the transcriptome analysis (Supplementary Figure S3).

## Discussion

### The Anthocyanin Content and Color Variation between ‘Red Bartlett’ and ‘Starkrimson’

The formation of red-skinned pears is dependent on the level of anthocyanin in the fruit and its differing accumulation patterns. In some European pears, the peak of red fruit coloration appeared in the middle development stage and faded at harvest (Steyn et al., 2004). In ‘Red Bartlett,’ anthocyanin levels peaked at 56 DAFB before fading gradually from 63 DAFB to maturation (Wang et al., 2013). In this study, ‘Red Bartlett’ and ‘Starkrimson’ had similar pigmentation patterns and showed higher anthocyanin levels early in fruit development that then peaked in the middle of the development stage. The anthocyanin level then significantly decreased in ‘Red Bartlett’ fruit after 55 DAFB but did not change significantly in ‘Starkrimson,’ consistent with the observed changes in fruit color of ‘Red Bartlett’ and ‘Starkrimson’ during development (**Figure 1**). These results indicate that the color fading of fruit skin late in the development of ‘Red Bartlett’ pears is induced by decreasing anthocyanin levels.

### DEGs Involved in the Anthocyanin Biosynthesis Pathway

Anthocyanin biosynthesis relies on the transcript levels of a series of encoding structural genes, most of which have been isolated and cloned in pear (Zhang et al., 2011; Yang et al., 2015b). In this study, transcriptome analysis showed that the expression level of two LDOX genes significantly decreased in the later stages of fruit development in ‘Red Bartlett.’ UFGT, a key anthocyanin biosynthesis gene, decreased in expression level at 75 DAFB in ‘Red Bartlett’ (**Figure 4A**). This gene was not one of the 947 DEGs selected for further analysis, and may not be a determinant of the color variation of the two pears. Therefore, we speculated that LDOX was closely related to color fading of ‘Red Bartlett’ and that UFGT was not. FLS may be involved in the flavonol pathway (Holton et al., 1994) and its expression was found here to be significantly upregulated in the later stages of ‘Red Bartlett’ fruit development. FLS expression levels were negatively associated with anthocyanin accumulation. These results demonstrate that the reduced anthocyanin levels in ‘Red Bartlett’ may be associated with decreased anthocyanin synthesis and increased FLS.

Anthocyanin biosynthesis is usually regulated by various TFs and in particular the MBW complex (Gonzalez et al., 2008). By analyzing RNA-Seq data, many genes encoding TFs were identified, including seven MYBs (**Figure 4B**), four of which were significantly downregulated in transcription in the late development stage of ‘Red Bartlett’ while the others were upregulated. Previously, genes like MYB10 and its homolog MdMYB110a (Lee and Choo, 2013) were found to be key activators of the anthocyanin biosynthetic pathway in Rosaceae (Lin-Wang et al., 2010). In this study an MYB10 homolog (LOC103959467) showed a significant decrease in transcription from the early to late stages in ‘Red Bartlett,’ suggesting that MYB10 expression correlates with anthocyanin levels. However, LOC103959467 was not significantly differentially expressed between the two cultivars at 75 DAFB and, therefore, did not belong to the group of 947 redundant DEGs. This suggests that the most important factor responsible for the color fading of ‘Red Bartlett’ pears could not be MYB10. Transcript levels of other MYBs were significantly reduced during color fading in ‘Red Bartlett,’ indicating a positive correlation with anthocyanin accumulation. Additionally, The co-expression of MYB10 and bHLH3 activated expression of dihydroflavonol-4-reductase and UFGT, leading to anthocyanin accumulation (Xie et al., 2012; Ravaglia et al., 2013). In our study, five bHLHs were upregulated and three were downregulated during the later development stage of ‘Red Bartlett’ (**Figure 4B**). Furthermore, in the late development stage of ‘Red Bartlett,’ expression of bHLH3 (LOC103947578) was significantly upregulated, while expression of bHLH33 (LOC103932269) was significantly downregulated. These results suggest that the bHLH family is involved in the regulation of anthocyanins synthesis by the different expression pattern.

Recently, the connections between other TFs and anthocyanin synthesis have attracted much attention. A gene encoding an NAC protein was found to be highly upregulated in blood-fleshed peach (Zhou et al., 2015). The overexpression of *zinc finger of Arabidopsis 6* inhibited root growth and increased anthocyanin accumulation (Devaiah et al., 2007). In the present study, eight WRKY, nine ERF, four NAC, and nine zinc finger TFs were also screened from the 947 DEGs, with 12 found to be upregulated and 18 downregulated during the color fading stages of ‘Red Bartlett’ (**Supplementary Table S5**). These genes may be involved in the regulation of anthocyanin accumulation. Also, these genes point out the direction for further study on coloration of fruit.

### DEGs Involved in Anthocyanin Transport and Degradation

In plants, GSTs were involved in the transfer of anthocyanins from the endoplasmic reticulum to the vacuole and were required for anthocyanin sequestration (Marrs et al., 1995; Mueller et al., 2000). Previously, GST showed a positive correlation with anthocyanin accumulation, such as TT19 (Kitamura et al., 2004). According to our results, transcription of six GST genes decreased significantly in the late development stages of ‘Red Bartlett’ fruit (**Figure 4A**), corresponding to the reduction in anthocyanin levels. So down-regulated GSTs may played a negative role in anthocyanin transport and led to a decrease in vacuolar anthocyanin levels. In addition, the ABC-C transporter and MATE family were also involved in the transport and accumulation of anthocyanins (Goodman et al., 2004; Gomez et al., 2011). In this study, two MATE genes were significantly downregulated at the later stages of ‘Red Bartlett’ fruit development. These results suggest that expression of the two MATE genes correlated positively with anthocyanin accumulation. Thus, anthocyanin transport is closely linked to color fading of ‘Red Bartlett’ pears.

Vacuolar peroxidases, a class III peroxidase family, were more likely candidates for anthocyanin degradation in plants (Ferreres et al., 2011), with this relationship confirmed in Brunfelsia (Vaknin et al., 2005; Zipor et al., 2015). Later, an intracellular laccase, an anthocyanin degradation enzyme, was found to be responsible for epicatechin-mediated anthocyanin degradation in litchi fruit pericarp (Fang et al., 2015). In the present study, one LAC gene and two POD genes were identified among the 947 DEGs (**Figure 4A**). The expression of the three genes increased significantly at 75 DAFB in ‘Red Bartlett’ as the anthocyanin content of the fruit peel decreased significantly. This suggests that increased anthocyanin degradation was likely to contribute to the color fading of ‘Red Bartlett’ pears.

### DEGs Related to Light Signal

The light signal usually activated a series of anthocyanin-associated structural and regulatory genes that control anthocyanin synthesis (Cominelli et al., 2008; Guan et al., 2015). In our study, GO annotation and KEGG pathway enrichment analysis determined that the 947 DEGs were significantly enriched in the ‘Photosynthesis’ and ‘Circadian rhythm’ pathways (**Figure 3** and **Supplementary Table S3**). In particular, the module ‘Circadian rhythm’ included several genes responding to light signals, such as phytochrome B,Phytochrome-interacting factors 3, SPA, and COP1. These genes significantly increased in expression during the color fading stage of ‘Red Bartlett’ and negatively correlated with anthocyanin accumulation (**Supplementary Table S6**). In darkness, by the way of ubiquitination, the COP1/SPA complex was responsible for degradation of light-induced TFs involved in the regulation of anthocyanin synthesis, such as R2R3-MYBs (Zhu et al., 2008; Li et al., 2012; Maier et al., 2013). In our study, the COP1/SPA complex was upregulated significantly in the later development stage of ‘Red Bartlett’ pears. This results in decreased anthocyanin biosynthesis through the action of anthocyanin degrading regulatory factors and leads to a reduction in the level of anthocyanin in ‘Red Bartlett.’ These data suggest that the light signal may played a crucial role in the regulation of anthocyanin synthesis, confirming that the light signal was closely related to color fading in the fruit skin of ‘Red Bartlett’ pears.

## Conclusion

**FIGURE 5:**
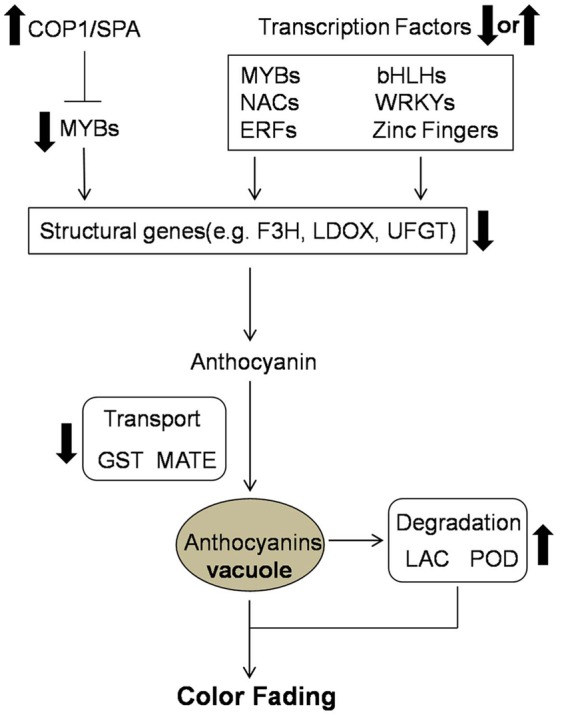
**Predicted profile of the color fading of ‘Red Bartlett’.** Thick arrows indicate upregulation or downregulation in the late fruit development of ‘Red Bartlett’.

Candidate genes involved in the color fading of ‘Red Bartlett’ pears were identified using transcriptome analysis. Based on our results, the color fading of ‘Red Bartlett’ was a complex process regulated by a series of metabolic pathways (**Figure 5**). The light signal was perceived by the light receptor and maybe involved in anthocyanin synthesis by regulating TFs. Furthermore, MYB, bHLH, WRKY, NAC, ERF, and zinc finger TFs were more likely to have a tight connection with anthocyanin accumulation. In anthocyanin transport, GST and MATE were significantly down-regulated in 75 DAFB of ‘Red Bartlett,’ indicating a decrease in anthocyanin accumulation in the vacuole. Finally, anthocyanin degradation enzymes like LAC and POD might play a critical role in degrading anthocyanin.

## Author Contributions

ZW and LX: conceived the project; RZ and LS: carried out experiments; ZW, RZ, and HD: analyzed the data; ZW, HD, FM, LX: manuscript preparation and editing.

## Conflict of Interest Statement

The authors declare that the research was conducted in the absence of any commercial or financial relationships that could be construed as a potential conflict of interest.

## Footnotes

1 https://www.ncbi.nlm.nih.gov/sites/batchentrez

## Supplementary Material

TABLE S1 **List of RT-qPCR primers**.

TABLE S2 **Summary of 947 DEGs in RNA-Seq**.

TABLE S3 **KEGG pathways of 947 DEGs in RNA-Seq**.

TABLE S4 **Differentially expressed genes involved in the anthocyanin anthocyanin synthesis, degradation, and transport**.

TABLE S5 **Differentially expressed genes involved in the anthocyanin regulation**.

TABLE S6 **Differentially expressed genes response to light signal**.

TABLE S7 **Statistics of RNA-Seq data**.
