# Supplementary material for: Transcriptome Analysis Reveals Candidate Genes Related to Color Fading of ‘Red Bartlett’ (Pyrus communis L.)
Source: Front Plant Sci. 2017 Mar 31;8:455. doi: 10.3389/fpls.2017.00455 (PMC5374147; doi:10.3389/fpls.2017.00455)
Supplement: Supplementary file 2 [file Data_Sheet_2.docx]

**Supplementary Material**

**Transcriptome analysis reveals candidate genes related to color fading of ‘Red Bartlett’ (*Pyrus communis* L.)**

Zhigang Wang^†^, Hong Du^†^, Rui Zhai, Linyan Song, Fengwang Ma, Lingfei Xu*

College of Horticulture, Northwest A&F University, Yangling, Shaanxi Province, China

*** Correspondence:**Lingfei Xu
email：lingfxu2013@sina.com

† These authors contributed equally to this work.

**Supplementary Figure**

**Figure S1.** Expression of ACS1 gene.

**Figure S2.** Gene coverage from RNA-Sequencing (RNA-Seq) data. Four RNA-Seq libraries were constructed using the fruit skin of Starkrimson-35 (35 days after full bloom (DAFB) of ‘Starkrimson’), Red Bartlett-35 (35 DAFB of ‘Red Bartlett’), Starkrimson-75 (75 DAFB of ‘Starkrimson’), and Red Bartlett-75 (75 DAFB of ‘Red Bartlett’), respectively.

**Figure S3.**Validation of the relative expression level of candidate genes related to the color fading phenotypeof ‘Red Bartlett’. (A) Validation of the relative expression level of candidate genes in ‘Starkrimson’.(B) Validation of the relative expression level of candidate genes in ‘Red Bartlett’. The vertical axis shows the value of log_2_ (Fold Change, FC), while the horizontal axis representsLOC103954960 (DFR), LOC103958614 (LDOX), LOC103951514 (UFGT), LOC103959467 (MYB10), LOC103956865 (MYB108-Like), LOC103961264 (MYB108), LOC103961604 (MYB44-Like), LOC103947578 (bHLH3), LOC103932269 (bHLH33), LOC103944005 (bHLH041), LOC103960192 (GST) and LOC103961180 (POD), respectively.


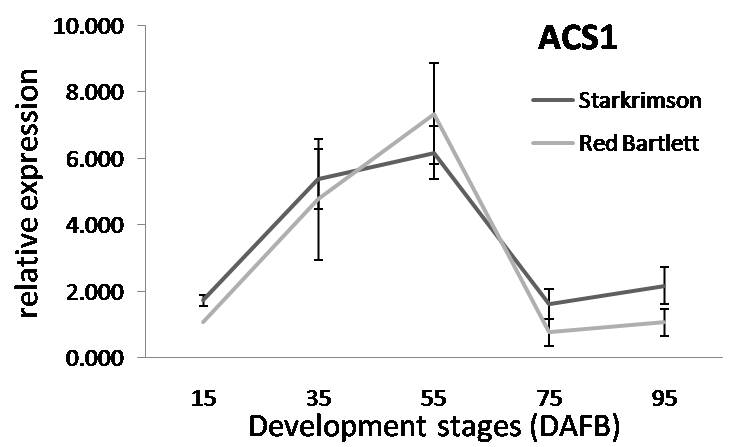


**Figure S1.** Expression of ACS1 gene.

**
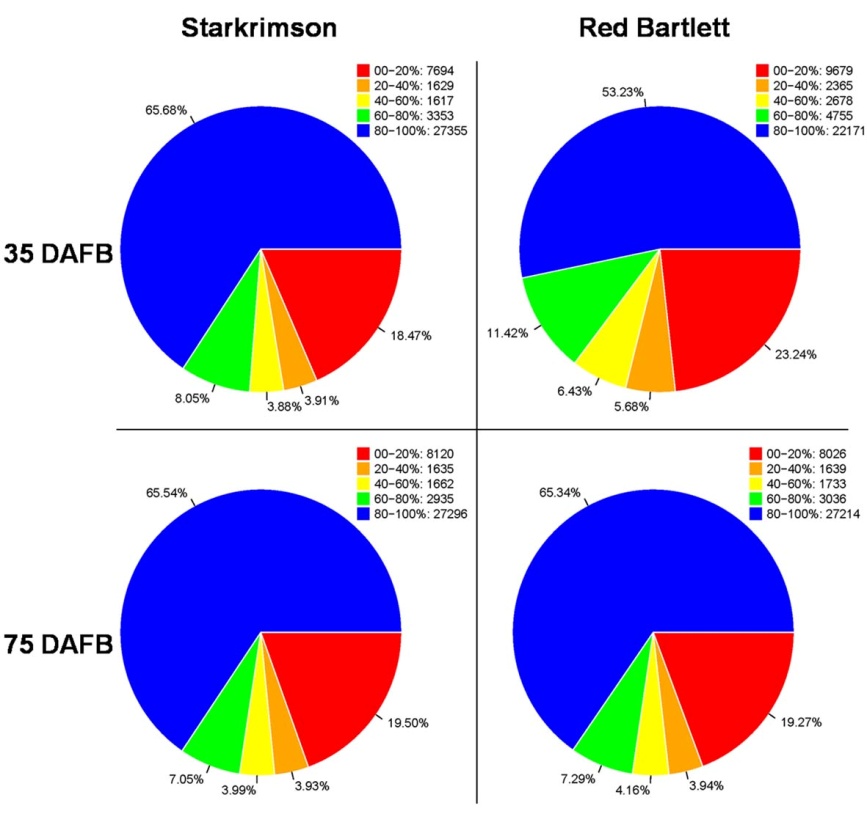
**

**Figure S2.** Gene coverage from RNA-Sequencing (RNA-Seq) data. Four RNA-Seq libraries were constructed using the fruit skin of Starkrimson-35 (35 days after full bloom (DAFB) of ‘Starkrimson’), Red Bartlett-35 (35 DAFB of ‘Red Bartlett’), Starkrimson-75 (75 DAFB of ‘Starkrimson’), and Red Bartlett-75 (75 DAFB of ‘Red Bartlett’), respectively.


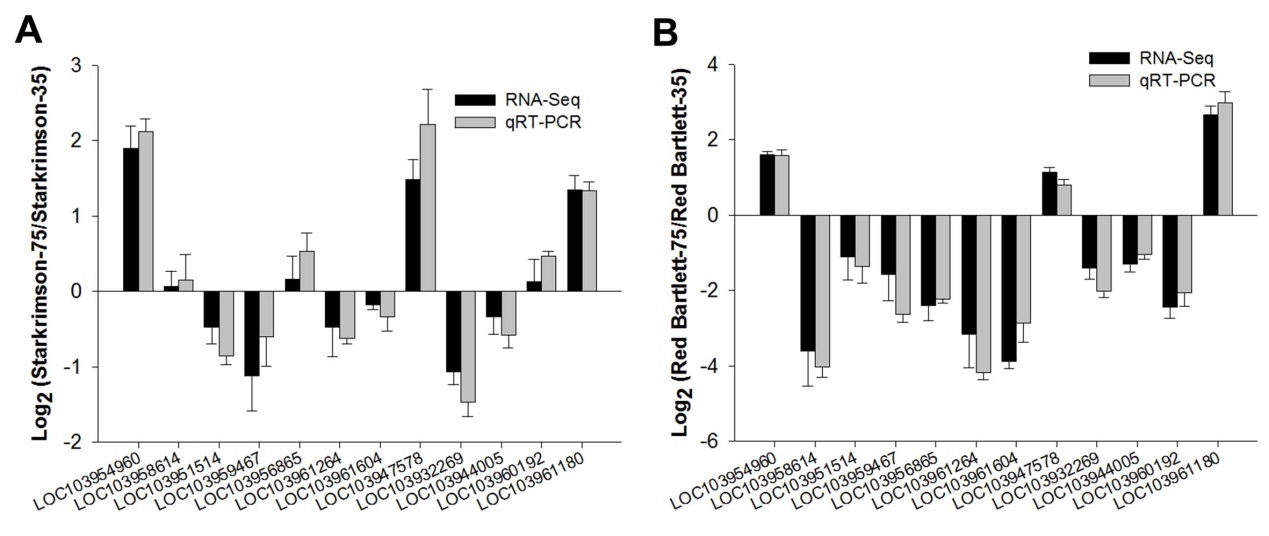


**Figure S3.** Validation of the relative expression level of candidate genes related to the color fading phenotypeof ‘Red Bartlett’. (A) Validation of the relative expression level of candidate genes in ‘Starkrimson’.(B) Validation of the relative expression level of candidate genes in ‘Red Bartlett’. The vertical axis shows the value of log_2_ (Fold Change, FC), while the horizontal axis representsLOC103954960 (DFR), LOC103958614 (LDOX), LOC103951514 (UFGT), LOC103959467 (MYB10), LOC103956865 (MYB108-Like), LOC103961264 (MYB108), LOC103961604 (MYB44-Like), LOC103947578 (bHLH3), LOC103932269 (bHLH33), LOC103944005 (bHLH041), LOC103960192 (GST) and LOC103961180 (POD), respectively.
